# A predictive model for postoperative progressive haemorrhagic injury in traumatic brain injuries

**DOI:** 10.1186/s12883-021-02541-w

**Published:** 2022-01-07

**Authors:** Tiange Chen, Siming Chen, Yun Wu, Yilei Chen, Lei Wang, Jinfang Liu

**Affiliations:** 1 Department of Neurosurgery, Xiangya Hospital, Central South University, No.87 Xiangya Road, Changsha, Hunan 410008 People’s Republic of China; 2 Department of Anesthesiology, Xiangya Hospital, Central South University, Changsha, Hunan China

**Keywords:** Nomogram, Progressive haemorrhagic injury, Traumatic brain injury

## Abstract

**Background:**

Progressive haemorrhagic injury after surgery in patients with traumatic brain injury often results in poor patient outcomes. This study aimed to develop and validate a practical predictive tool that can reliably estimate the risk of postoperative progressive haemorrhagic injury (PHI) in patients with traumatic brain injury (TBI).

**Methods:**

Data from 645 patients who underwent surgery for TBI between March 2018 and December 2020 were collected. The outcome was postoperative intracranial PHI, which was assessed on postoperative computed tomography. The least absolute shrinkage and selection operator (LASSO) regression model, univariate analysis, and Delphi method were applied to select the most relevant prognostic predictors. We combined conventional coagulation test (CCT) data, thromboelastography (TEG) variables, and several predictors to develop a predictive model using binary logistic regression and then presented the results as a nomogram. The predictive performance of the model was assessed with calibration and discrimination. Internal validation was assessed.

**Results:**

The signature, which consisted of 11 selected features, was significantly associated with intracranial PHI (*p* < 0.05, for both primary and validation cohorts). Predictors in the prediction nomogram included age, S-pressure, D-pressure, pulse, temperature, reaction time, PLT, prothrombin time, activated partial thromboplastin time, FIB, and kinetics values. The model showed good discrimination, with an area under the curve of 0.8694 (95% CI, 0.8083–0.9304), and good calibration.

**Conclusion:**

This model is based on a nomogram incorporating CCT and TEG variables, which can be conveniently derived at hospital admission. It allows determination of this individual risk for postoperative intracranial PHI and will facilitate a timely intervention to improve outcomes.

## Background

Nearly 50 million people worldwide suffer from traumatic brain injury (TBI) each year [1]. According to a study by the Global Burden of Disease, the prevalence of TBI increased by 8.4% between 1990 and 2016 [2].C,oagulopathy is a common complication of TBI and is associated with an increased morbidity and mortality [3]; it has an estimated incidence of 7–63% [4, 5]. Coagulopathy is often the main cause of postoperative progressive haemorrhagic injury (PHI) [6, 7]. For patients who have undergone surgery after TBI, intracranial PHI often leads to increased intracranial pressure and cerebral herniation, further threatening the patients’ lives. Studies have shown that the mortality rate of patients with PHI is nine times higher that of patients without PHI [8]. The incidence of PHI in patients with craniocerebral trauma is approximately 23% [9, 10].

Predicting the outcome of this condition is challenging because of the complex mechanism of coagulopathy and the lack of uniform diagnostic and evaluation criteria. Routine laboratory indicators for clinical detection of coagulopathy mainly include prothrombin time (PT), activated partial thromboplastin time (APTT), international normalised ratio (INR), and platelet count, jointly called conventional coagulation test (CCT) findings. Although widely used, CCTs have several limitations. First, the determination of platelet count and fibrinogen can only reflect quantity, not function. Second, PT, APTT, and INR can reflect the disturbance of a single pathway but cannot be used to assess the complex interaction between multiple pathways and the overall coagulation function [11].Many hospitals also use thromboelastography (TEG) for the clinical detection of coagulation diseases; the measures used in TEG include reaction time (R), kinetics time (K), α angle, maximum amplitude (MA), percent lysis 30 min after MA, and coagulation index. As a measure of clot strength, TEG does not rely on biochemical pathways affecting coagulation [12]. Windelov et al. showed that TEG was more effective than CCT in predicting poor prognosis in patients with craniocerebral trauma [13]. Other studies have shown that TEG can provide information on coagulation function faster than CCT can, thus enabling timely correction of coagulation dysfunction in patients with craniocerebral trauma [14, 15].

Therefore, CCT together with TEG can effectively reflect the quantity and quality of coagulation factors and platelets, reflect the speed and intensity of clot formation, and, thus, reflect the overall coagulation function. A clinical prediction model based on CCT and TEG could enable clinicians to intuitively understand the coagulation function of patients with craniocerebral trauma, provide a basis for component blood transfusion, reduce occurrence of postoperative PHI, and improve the prognosis of patients. To this end, we developed and validated a clinical prediction model to support the management of postoperative coagulopathy in patients with TBI.

## Methods

### Patients

We collected the clinical medical records and imaging data of 645 TBI patients admitted to the Department of Neurosurgery, Xiangya Hospital, Central South University from March 2018 to December 2020 who met the following inclusion criteria: (1) a clear history of head trauma, (2) age ≥ 18 years, (3) hospital admission within 72 h after injury, and (4) need for surgical treatment. The exclusion criteria were: (1) use of antiplatelet (such as aspirin and clopidogrel) or anticoagulant drugs (such as warfarin); (2) severe multiple organ failure; (3) associated hematologic system diseases; (4) hemodynamic instability during admission to the intensive care unit (heart rate < 50 beats/min or systolic blood pressure < 90 mmHg or mean arterial pressure < 65 mmHg); (5) severe multiple injuries (ISS ≥ 25 points); (6) previous central nervous system diseases (such as stroke and brain tumour); (7) pregnancy; and (8) incomplete clinical data.

### Data extraction

The clinical data, including demographic characteristics, Glasgow Coma Scale (GCS) score upon admission, pupil diameter, light reflex, and postoperative CCTs and TEG findings, were extracted from the hospital’s case management system.

### Statistical analysis

All statistical analyses were conducted using the R software (version 4.0.3; https://www.Rproject.org). Demographic data of the patients were evaluated by T test and X^2^ test. For continuous variables, t-test is used for comparison, and for categorical variables, X^2^ test is used. The reported statistical significance levels were all two-sided, with statistical significance set at 0.05.

### Outcome measures

All patients underwent a computed tomography (CT) scan within 6 h postoperatively to determine if there was any bleeding in the surgical area. Subsequently, CT scans were performed from 6 h after surgery to the time of discharge as needed to determine whether PHI had occurred.

Our outcome was postoperative intracranial PHI, which was defined as a intracranial haemorrhage on any subsequent postoperative computed tomography (CT) scan that was not seen in the initial scan or the amount of bleeding in the original site was 25% higher than that in the previous CT scan [16, 17]. We divided the patients into two groups according to the above criteria, namely PHI group and non-PHI group.

### Predictor variables

We screened several features from 16 candidate predictors (i.e. age, gender, S-pressure, D-pressure, pulse, temperature, GCS score, R, K, αangel, MA, PLT, PT, APTT, FIB), which were consistently associated with the outcome of PHI, using the least absolute shrinkage and selection operator (LASSO) binary logistic regression model, univariate analysis, and the Delphi method for inclusion in the prediction models [18]. We log transformed values for continuous variables, such as temperature and K.

### Model development

The association between predictor variables and outcomes was analysed using logistic regression models. Prognostic strength was quantified as odds ratios (ORs) with 95% confidence intervals (CIs). In order to facilitate clinical application, we established a nomogram based on binary logic analysis. Receiver operating characteristic (ROC) curves were drawn to evaluate the predictive value of the model.

### Model performance

An ROC curve was plotted using R software, and the ability of the model to discriminate between patients with PHI and without PHI was assessed using an area under the receiver operator characteristics (AUC) curve analysis. A perfect model would have an AUC of 1.

### Model validation

Internal validation evaluates the stability of a prediction model against random changes in sample composition. Due to limitation of samples, external validation is not appropriate in our study. Internal validation was performed using the bootstrap resampling technique, where regression models were fitted in 100 bootstrap replicates drawn with replacement from the development sample. The model was refitted in each bootstrap replicate and tested on the original sample to estimate optimism in model performance.

All methods were performed in accordance with the TRIPOD statement published in 2015 [19].

## Results

### Clinical characteristics

**Fig. 1:**
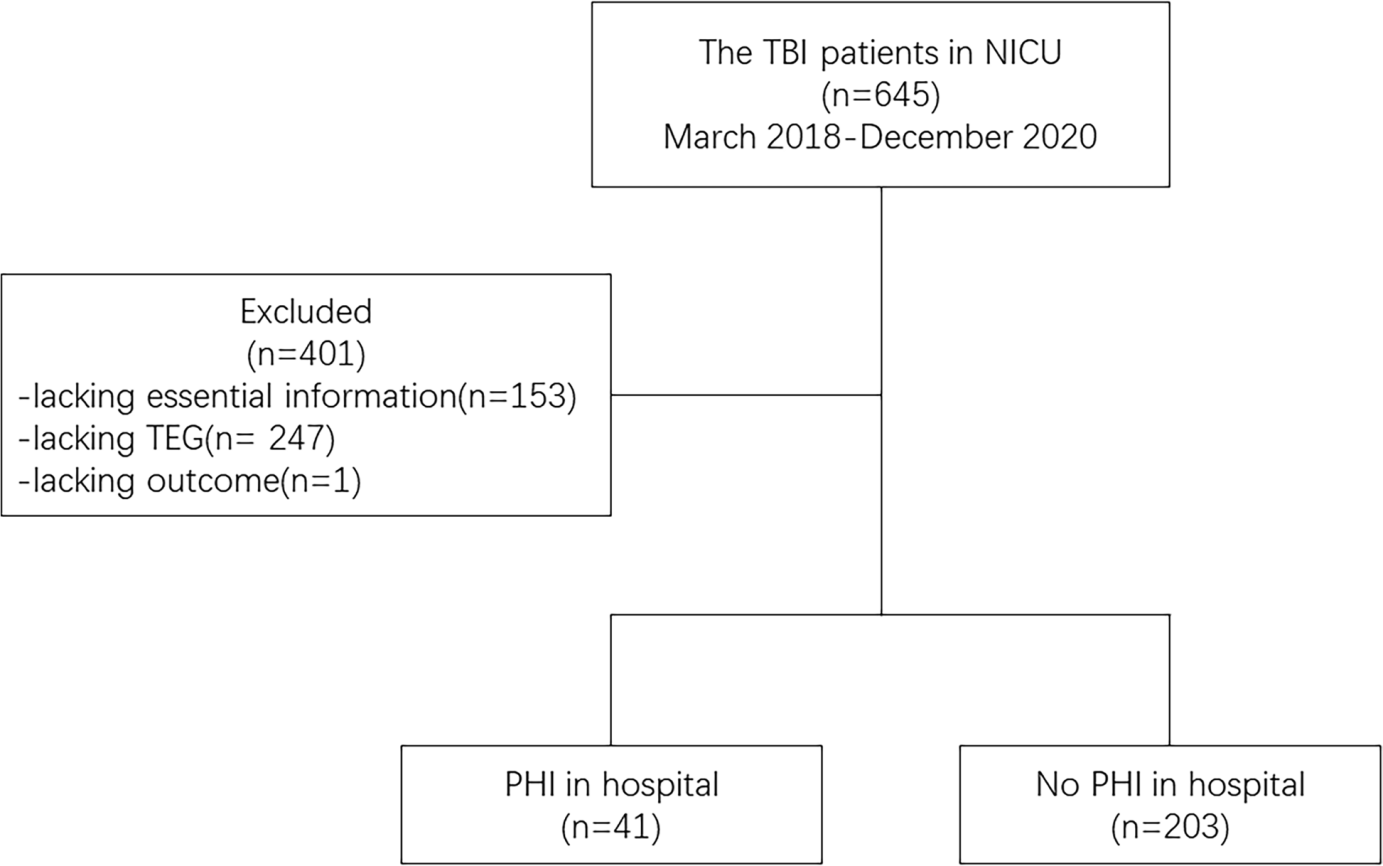
Screening of the patient cohort for the development of the prediction model

Patient characteristics are shown in Table 1. We collected data of 645 patients treated in the Neurosurgical intensive care unit of Xiangya Hospital. A total of 153 patients and 247 patients were excluded because of lack of TEG, PT, APTT tests data information, respectively. One patient was excluded because outcome data was not available (Fig. 1). In total, 203 patients did not suffer intracranial PHI after surgery, but it was observed in 41 patients. There was no statistical difference in the distributions of age, sex, D-pressure, pulse, temperature, GCS score, R value, K value, angle, and MA between the two groups (PHI group and non-PHI group). S-pressure (*p =* 0.04), PLT (*p* < 0.001), APTT (*p* < 0.001), and FIB (*p* = 0.003) were significantly associated with postoperative intracranial PHI.

**Table 1:** Demographic and clinical characteristics of the study population

Characteristic	PHI (+)	PHI (−)	***p-***value
**Age, mean ± SD, years**	54.90 ± 12.73	52.08 ± 13.90	0.243
**Gender, No. (%)**			0.869
**Male**	31 (75.61%)	151 (74.38%)	
**Female**	10 (24.39%)	52 (25.62%)	
**S-pressure, median (IQR)**	134.0 (118.5–155.5)	129.0 (117.0–134.0)	0.040
**D-pressure, median (IQR)**	77.0 (69.5–85.0)	76.0 (70.0–80.0)	0.591
**Pulse, median (IQR)**	80.0 (70.0–90.5)	80.0 (75.0–90.0)	0.649
**Temperature, median (IQR)**	37.0 (36.7–37.3)	37.0 (36.5–37.2)	0.331
**GCS score, median (IQR)**	8 (6–13)	9 (7–13)	0.354
**R, median (IQR)**	6.3 (5.4–7.3)	6.2 (5.1–7.3)	0.329
**K, median (IQR)**	1.5 (1.3–2.0)	1.6 (1.2–2.2)	0.695
**α angel, median (IQR)**	67.4 (60.5–71.7)	67.6 (60.8–72.2)	0.579
**MA, median (IQR)**	68.7 (60.1–75.3)	66.9 (61.4–72.5)	0.342
**PLT, median (IQR)**	104.0 (84.5–143.5)	149.0 (111.0–205.0)	<0.0001
**PT, median (IQR)**	16.2 (15.1–17.0)	14.3 (13.4–15.5)	<0.0001
**APTT, median (IQR)**	38.3 (34.5–43.7)	32.60 (29.1–38.4)	<0.0001
**FIB, median (IQR)**	2.7 (2.1–3.9)	3.7 (2.6–4.9)	0.003
**INR, median (IQR)**	1.3 (1.2–1.3)	1.1 (1.1–1.2)	<0.0001

*APTT* activated partial thromboplastin time, *FIB* fibrinogen, *GCS* Glasgow Coma Scale, *IQR* interquartile range, *INR* international normalised ratio, *K* kinetics time, *MA* maximum amplitude, *PHI* progressive haemorrhagic injury, *PLT* platelets, *PT* prothrombin time, *R* reaction time, *SD* standard deviation

### Feature selection

**Fig. 2:**
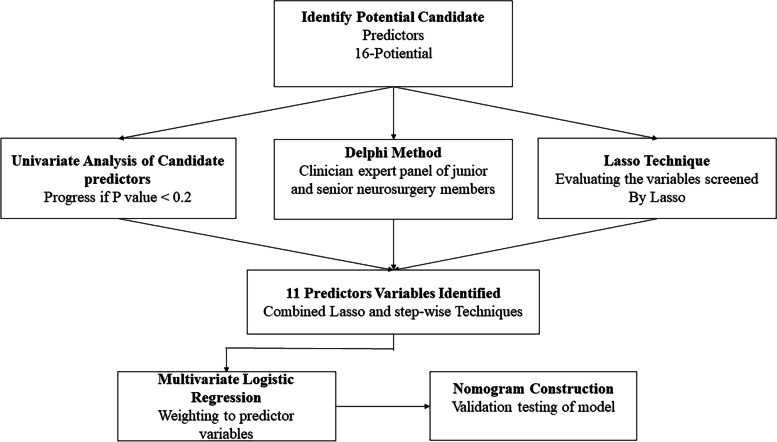
Feature selection using the least absolute shrinkage and selection operator binary logistic regression model, while the univariate Analysis and Delphi method are used for potential predictor selection

**Fig. 3:**
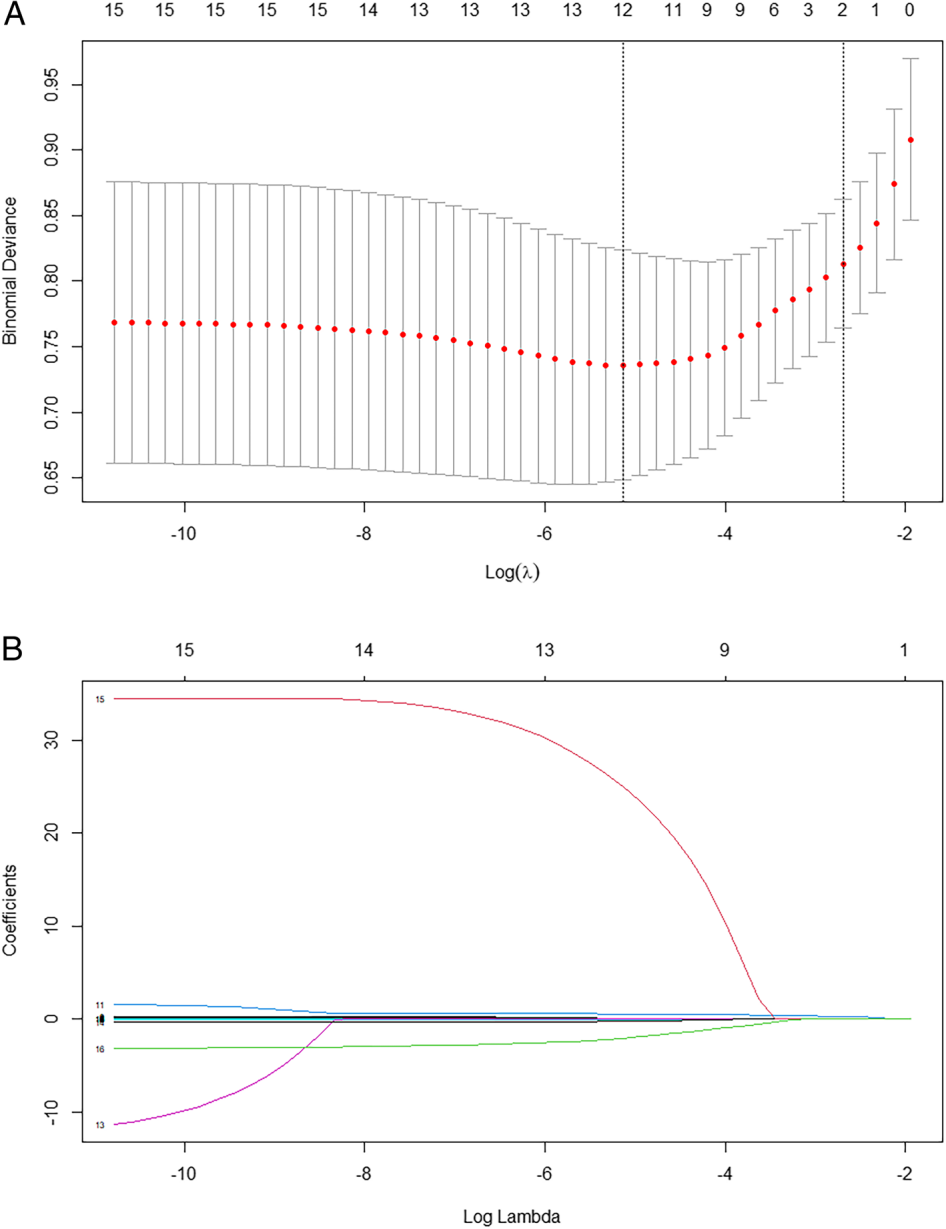
Feature selection using the least absolute shrinkage and selection operator (LASSO) binary logistic regression model. **A** Tuning parameter (λ) selection in the LASSO model using 10-fold cross-validation via minimum criteria. The area under the receiver operating characteristic (AUC) curve was plotted against log (λ). Vertical lines were drawn at the optimal values using the minimum criteria and one standard error of the minimum criteria (the 1-SE criteria). A value of 0.004 with log (λ) of − 5.324 was chosen (1-SE criteria) according to the 10-fold cross-validation. **B** LASSO coefficient profiles of 16 texture features. A coefficient profile plot was plotted against the log (λ) sequence. Using 10-fold cross-validation, the optimal λ resulted in 11 non-zero coefficients

In total, 16 candidate features were extracted from TEG and CCT variables. Of these, 11 potential predictors were selected on the basis of 244 patients in the cohort (Fig. 2). The predictors were selected by the LASSO logistic regression model were non-zero coefficients (Fig. 3). Based on the advice of experienced professor of neurosurgeries and researchers at our institution, 16 variables were evaluated using the Delphi method; from these, the most relevant clinical predictors were selected.

Based on the univariate analysis, Delphi method and Lasso technique results, 11 final predictors were identified: age, S-pressure, D-pressure, pulse, temperature, R, PLT, PT, APTT, FIB and K value (Table 2).

**Table 2:** Logistic regression analysis of clinical candidate predictors in the training set

Variable	OR (95% CI)	***p-***value
**Age**	1.020 (0.986–1.054)	0.250
**S-pressure**	1.041 (1.012–1.071)	0.005
**D-pressure**	0.971 (0.927–1.018)	0.224
**Pulse**	0.966 (0.937–0.996)	0.028
**Temperature**	2.407 (1.120–5.172)	0.024
**R**	1.109 (0.867–1.418)	0.412
**K**	0.375 (0.214–0.658)	0.001
**PLT**	0.989 (0.980–0.998)	0.013
**PT**	1.904 (1.361–2.664)	< 0.001
**APTT**	1.046 (0.979–1.117)	0.184
**FIB**	0.756 (0.524–1.091)	0.135

*APTT* activated partial thromboplastin time, *FIB* fibrinogen, *K* kinetics time, *PLT* platelet, *PT* prothrombin time, *R* reaction time

### Model development

**Fig. 4:**
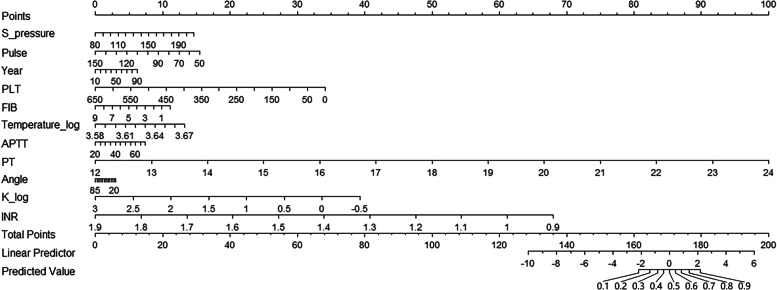
Nomogram developed for the prediction of postoperative intracranial progressive haemorrhagic injury in cases of traumatic brain injury

A model incorporating age, S-pressure, D-pressure, pulse, temperature, R, PLT, PT, APTT, FIB, and K value was developed and presented as a nomogram (Fig. 4).

### Performance assessment

**Fig. 5:**
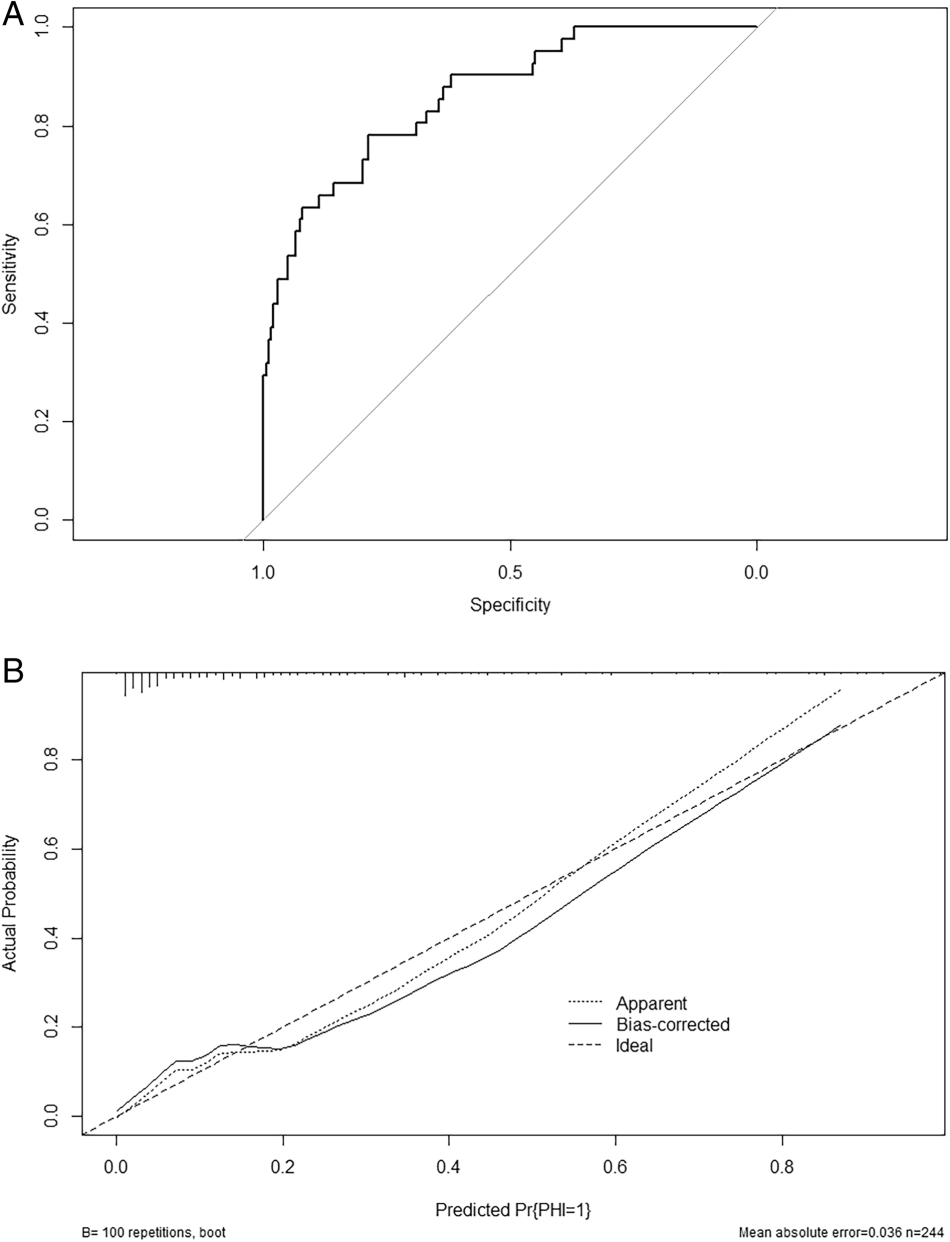
**A** Receiver operating characteristic curves of the nomogram. **B** Calibration curves of the nomogram. Illustration of the agreement between the predicted risk of postoperative intracranial PHI(progressive haemorrhagic injury) and the observed outcomes of postoperative PHI in patients with traumatic brain injury

Good discrimination (Fig. 5A) and good calibration (Fig. 5B) were observed in the validation set. The AUC of the nomogram was 0.8694 (95% CI, 0.8083–0.9304), and since it was > 0.75, it was considered to have excellent discrimination. In addition, the Hosmer-Lemeshow test also produced a nonsignificant *p-value* of 0.7233, which can prove the same thing [20].

### Clinical usefulness of the model

**Fig. 6:**
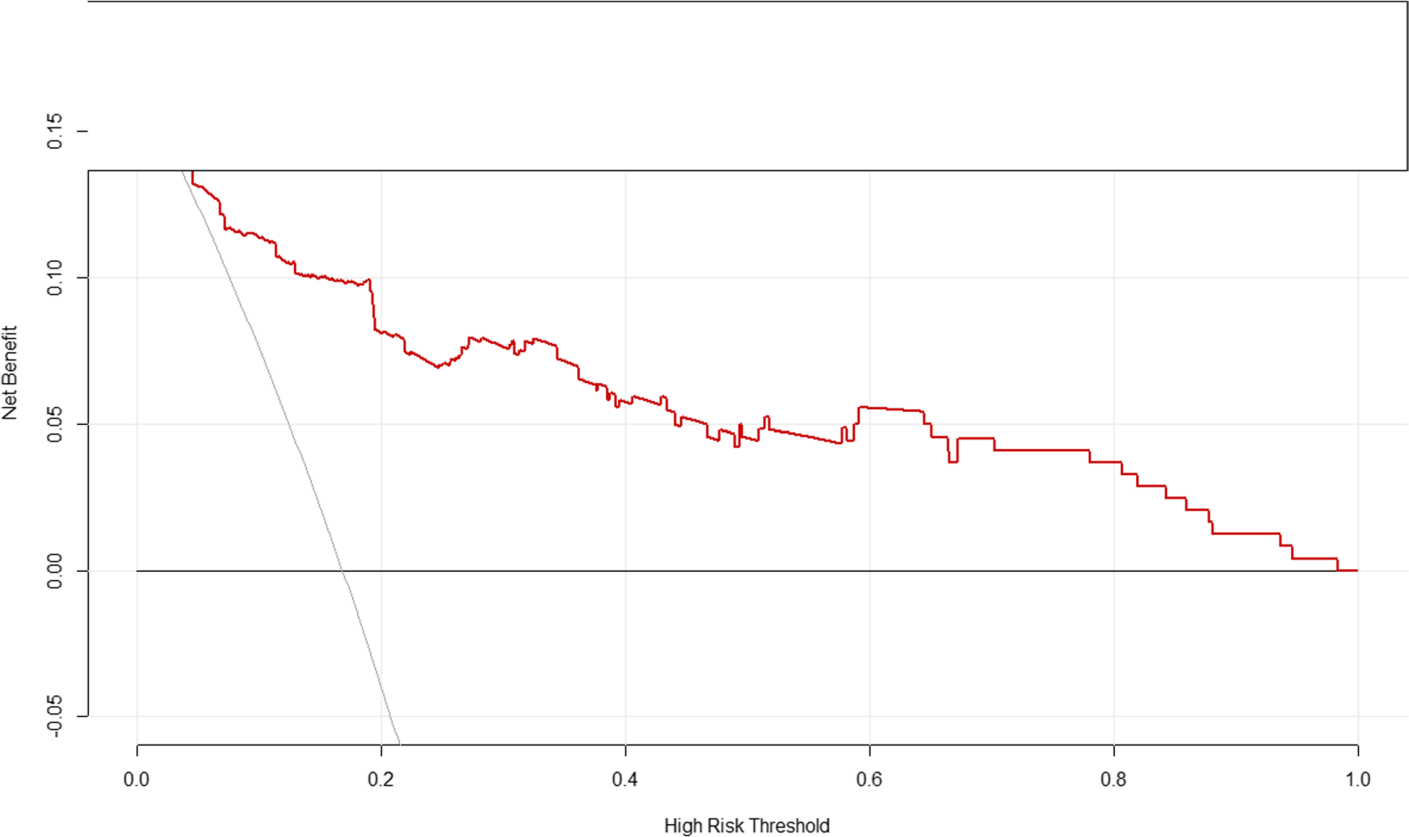
Decision curve analysis for the predictive model

The decision curve analysis (DCA) constructed by our data provided a net benefit over the “treat-all” or “treat-none” strategy at a high-risk threshold probability > 2.0% (Fig. 6), which show that the model is clinically useful. For instance, with a high-risk threshold probability of 40%, use of the model could provide an added net benefit of 0.268 compared to the “treat-all” or “treat-none” strategy.

## Discussion

To our knowledge, various of predictive models have been used to help clinician to evaluate the outcome of TBI patients [21]. However, this is the first study to develop and internally validate a tool to predict postoperative intracranial PHI in patients with TBI using CCTTEG-based clinically relevant predictors.

By intuitively predicting the risk of postoperative coagulation dysfunction in patients with TBI, this predictive model can identify patients with poor prognosis at early stages and allows timely clinical intervention, thus improving patient prognosis. We empirically define patient who gets a score in our model over 0.5 as high risk man and effective measures should been taken to prevent the intracranial PHI after surgery.

Our predictive model predicted the risk of postoperative intracranial PHI in TBI, which can facilitate early clinical interventions. There are two main measures to been taken to intervene the disorder of blood coagulation. The first one is component blood transfusion, including fresh frozen plasma, prothrombin complex concentrate, concentrated blood platelets, fibrinogen and reprogramming factors VII, which could supply the absence materials of blood and address the severe coagulopathy [22]. Another way is drug intervention. Many studies have suggested that TXA, desmopressin and other drugs can be used to improve coagulation dysfunction in patients with TBI [23, 24].

This study had some limitations. First, as till date, not enough TBI cases have been tested by TEG at our institution, there was no external validation of the model to examine its portability and generalizability. External validation can preclude the possibility of overfitting during modelling [25]. Therefore, a multicentre prospective clinical trial to validate or optimise this predictive model is warranted. Second, the retrospective nature of the study limited access to clinical data such as Abbreviated Injury Scale and illness severity scoring findings, which are used to comprehensively assess the severity of multiple injuries in the body that have an impact on the severity craniocerebral trauma. Third, the TBI patients included in this study were only treated with trepanation and drainage, which meant that patients with complex TBI requiring craniectomy were missing from this sample. Therefore, clinical data related to these patients were not included among the predictive variables in this study, which may limit the application of this model to complex TBI. Fourth, our conclusion is different with some published studies results that K-value and alpha angle were significant predictors,which is associated with severity of TBI [26, 27]. Finally, in this study, preoperative data on coagulation function status of patients were lacking and most of the patients underwent TEG only once after surgery, which may have led to individual differences having a negative impact on the development of our model.

## Conclusion

Our prediction model included a developmental set of 244 patients and achieved good performance in internal validation. As the predictor items, which have been previously identified as powerful prognostic indicators, in this prediction model are readily available at hospital admission, our model has potential for timely intervention.

## Data Availability

The datasets used and/or analysed during the current study are available from the corresponding author on reasonable request.

## Abbreviations

APTT: Activated partial thromboplastin time
CCT: Conventional coagulation test
GCS: Glasgow Coma Scale
INR: International normalised ratio
K: Kinetics time
LASSO: Least absolute shrinkage and selection operator
MA: Maximum amplitude
PHI: Progressive haemorrhagic injury
PT: Prothrombin time
R: Reaction time
TBI: Traumatic brain injury
TEG: Thromboelastography
PLT: Platelets
FIB: Fibrinogen
ISS: Injury severity score
